# Can perfluoroalkyl acids biodegrade in the rumen simulation technique (RUSITEC)?

**DOI:** 10.1186/s12302-015-0063-4

**Published:** 2015-11-23

**Authors:** J. Kowalczyk, S. Riede, H. Schafft, G. Breves, M. Lahrssen-Wiederholt

**Affiliations:** 1 Federal Institute for Risk Assessment, Max-Dohrn-Str. 8-10, 10589 Berlin, Germany; 2 Department Institute of Physiology, University of Veterinary Medicine Hannover, Foundation, Bischofsholer, Damm 15, 30173 Hannover, Germany

**Keywords:** Perfluoroalkyl acids, PFOS, PFOA, Biodegradation, Rumen microorganisms, RUSITEC

## Abstract

**Background:**

The behaviour of perfluoroalkyl acids (PFAAs) in tissues of ruminants has been shown to differ from that of monogastrics (J Agric Food Chem 61(12):2903–2912 doi:10.1021/jf304680j, 2013; J Agric Food Chem 62(28):6861–6870, 2014). This may be a consequence of the complex microbial ecosystem in the rumen. To evaluate this hypothesis, the recovery of PFAAs was studied using the rumen simulation technique as an indication for biodegradation in rumen. The PFAA-recovery from a microbial fermentation of feed containing PFAAs was compared to the same feed in the absence of ruminal microorganisms (MOs).

**Results:**

Release of PFAAs from feed into fermentation fluid was found to be faster for perfluorobutane sulfonic acid (PFBS) than for perfluorooctane sulfonic acid (PFOS). Differences between perfluoroalkyl carboxylic acids (PFCAs) could not be observed. Proportions of PFAAs recovered in the fermentation fluids decreased by increasing chain lengths for the perfluoroalkyl sulfonic acids (PFSAs) (31 % PFBS, 28 % perfluorohexane sulfonic acid [PFHxS], 20 % perfluoroheptane sulfonic acid [PFHpS], 11 % PFOS) and PFCAs (33 % perfluorohexane carboxylic acid [PFHxA], 32 % perfluoroheptane carboxylic acid [PFHpA], 24 % perfluorooctanoic acid [PFOA]). In contrast, levels in feed increased with increasing chain length for both PFSAs and PFCAs.

**Conclusion:**

The attachment of MOs to feed particles was assumed to account for higher PFAA levels in fermented feeds and for lower levels in the fermentation fluids. Total recovery of PFAAs was significantly lower in presence of ruminal MOs compared to experimental procedure under sterile conditions. Although, there are optimal reductive conditions for MOs in rumen, our results do not univocally indicate whether PFAAs were degraded by ruminal fermentation.

**Electronic supplementary material:**

The online version of this article (doi:10.1186/s12302-015-0063-4) contains supplementary material, which is available to authorized users.

## Background

Perfluoroalkyl acids (PFAAs) belong to the chemical group of per- and polyfluoroalkyl substances (PFAS) [3]. PFAS are used in numerous industrial applications (e.g. electroplating, fluoropolymer production, photography), fire extinguishing agents and as processing aids in impregnation agents for a large number of consumer products (e.g. textile, carpet and leather protectors and food contact papers) since the 1950s [4–6]. Because of their various applications, large amounts of PFAS have been released into the environment, leading to their presence and detection in wildlife and humans [7–12]. The most widely studied PFAAs are perfluoroalkyl carboxylic acids (PFCAs) and perfluoroalkyl sulfonic acids (PFSAs) with the most frequently detected eight-carbon homologues perfluorooctanoic acid (PFOA) and perfluorooctane sulfonic acid (PFOS). These substances have received most attention because of their persistence [3, 13], toxic effects to mammals [14] and their strong tendency for bioaccumulation [15] and biomagnification in food chain [16, 17]. The chemical and biological persistence of PFAAs is generally believed to be a result of the carbon–fluorine bond, which is one of the strongest covalent bonds in nature [18]. Concerns about the potential of toxicity, environmental persistence and bioaccumulation have led to restricting use and inclusion of PFOS and its related substances in the stockholm convention on persistent organic pollutions (POPs) in 2009 [19]. Also for PFOA, a stewardship agreement between the US environmental protection agency (US EPA) and the leading global manufacturers intends to eliminate the environmental emission until 2015 [20]. In the European union, the phase-out of long-chain PFCAs (C9, C11–C14), PFOA and its ammonium salt (APFO) is controlled using the European chemical regulation (REACH EC No. 1907/2006). These substances have been identified as substances of very high concern (SVHC) and are included into the candidate list as substances proposed for authorization. Moreover, PFOA is discussed for inclusion into the restriction proceedings regulated in REACH Annex XVII (Restriction, Art. 67) [21, 22].

Since PFAAs raised concern due to their high persistence, some studies have been performed to investigate methods for their complete decomposition [23–26]. Although various methods have shown to be more or less successful, they often are very energy-consuming, require extreme conditions or lead to by-product formation with unknown adverse effects [27].

The degradation of PFAAs in sewage sludge has been examined for wastewater treatment plants. The study [28] recognized that PFOA and PFOS disappeared quite rapidly under anaerobic reductive conditions (redox potential of −380 mV). A possible microbially-mediated transformation of PFOA was observed [29] during the reductive dechlorination of trichloroethene when sewage treatment plant samples were used as inoculum for in vitro incubation. However, transformation products could not be clearly identified, thus, PFOA biodegradation was not proven successful. Only one study was found that reports the degradation of PFOS under ambient conditions [30]. Ochoa-Herrera and coworkers demonstrated that PFOS defluorination was catalyzed by cobalamin and simultaneous presence of titanium(III)citrate as reducing agent to achieve corresponding reductive conditions [30]. The complete degradation of PFOS (mineralization) was confirmed by measurement of fluoride ion release during the reductive dehalogenation process. In this study cobalamin was shown to be an important biomolecule for the reductive dehalogenation of PFOS. In nature, cobalamin is frequently involved in reductive dehalogenase catalysis [31]. This essential biomolecule is required by animals and humans, but can only be synthesized by bacteria and archaea [32].

In cows, the rumen contains a variety of bacterial species able to produce cobalamin and its analogues [33]. In combination with the reductive condition of about −300 to −400 mV, the microbial fermentation in the rumen might provide good conditions for the degradation of PFAAs and could affect the PFAA-adsorption downstream.

Interestingly, transfer studies on dairy cows and fattening pigs showed a different behaviour of PFAAs regarding partitioning to blood plasma, liver and edible tissues [1, 2]. Both species were exposed to PFAAs of different chain-length through the same naturally contaminated hay. Although there was an ad libitum feeding of PFAA-contaminated hay for dairy cows (app. 14 % of diet) and a restrictive feeding of a PFAA-hay containing pelleted feed for fattening pigs (app. 17 % of diet), the results of the percent of PFAAs accumulation in the estimated mass of tissues are well comparable and indicate different toxicokinetics in both species. The results showed that PFBS and PFOA had no tendency of accumulation in blood plasma of dairy cows compared to PFHxS and PFOS, while the affinity for blood plasma were evenly high for all PFAAs in fattening pigs. Considering the PFAA accumulation in edible tissue, similar amounts of all examined PFAAs (40–49 %) were found in the meat of fattening pigs. This was in particular contrast to the different amounts of PFOS (43 %), PFOA (<1 %) and PFBS (<0.001 %) in the muscle tissues of the dairy cows [1, 2]. Furthermore, excretion of PFBS and PFOA via milk throughout the feeding study was very low (<1 %) and cannot explain the low affinity for muscle in dairy cows. Because of the different findings, it was assumed that the difference of toxikokinetics in ruminants and monogastrics might be the consequence of their species-specific digestive system. The hypothesis was put forth that the microbial fermentation in rumen of bovine species might have an effect on PFAA recovery. It was posited that ruminal MOs may have effects on PFAA degradation because numerous studies in ruminants with other undesirable substances in feed, such as secondary plant compounds, mycotoxins, but also antibiotics have demonstrated the capacity of ruminal microorganisms to degrade potentially toxic compounds and thus protecting the host from detrimental effects [34–36]. Overall, MOs in the rumen are presumed to have an unexpectedly high capacity to degrade or transform fluorinated compounds.

The aim of this work was to evaluate the PFAA recovery as an indication of microbial degradation of PFAAs in the rumen in standardized conditions by using the in vitro rumen simulation technique (RUSITEC). The in vitro technique was chosen due to the ability to analyze the PFAA concentration in the fermented feed and liquid fractions of rumen at different points in time during the ruminal fermentation. Besides PFAA recovery, the effects of PFAAs on the fermentation characteristics (e.g. pH value, redox potential) and on microbial digestion of feed nutrients were investigated.

## Result and discussion

### Fermentation characteristics

The chemical compositions of the PFAA and PFAA-free hay are shown in Table 1. The PFAA hay had higher levels of crude fiber (CF, 34.6 %) than the PFAA-free hay (30.8 %), indicating that the grass grown on a PFAA contaminated farmland was cut later than the non-contaminated grass. Differences in crude protein (CP) level between PFAA hay (7.07 %) and PFAA-free hay (18.78 %) were also observed.

**Table 1:** Ingredients of the fermentation vessels, chemical composition and PFAA concentration of the PFAA hay and the PFAA-free hay

Ingredients, g/vessel	PFAA feed			PFAA-free feed^c^
	Low dosage^a^		High dosage^b^	
PFAA hay	7.998 ± 0.002		16.001 ± 0.004	
PFAA-free hay		8.002 ± 0.002		15.999 ± 0.001
Concentrate*	2.000 ± 0.002	1.997 ± 0.003	3.997 ± 0.003	3.997 ± 0.002

	PFAA hay	PFAA-free hay		
Chemical composition, % DM				
Dry matter (DM, %)	92.37	88.90		
Crude ash (CA)	7.85	7.08		
Crude protein (CP)	7.07	18.78		
Crude lipids (CL)	1.11	1.69		
Crude fiber (CF)	34.62	30.82		
Nitrogen free extracts (NfE)	53.40	41.62		
PFAA concentration, µg/kg				
PFBS	779.0 ± 27.9	<LOD^d^		
PFHxA	235.6 ± 4.9	<LOD^d^		
PFHpA	37.7 ± 0.7	<LOD^e^		
PFHxS	489.0 ± 20.5	<LOD^e^		
PFOA	91.6 ± 1.4	<LOD^e^		
PFHpS	20.8 ± 0.3	<LOD^e^		
PFNA	0.6 ± 0.0	<LOD^e^		
PFOS	611.9 ± 9.1	<LOD^e^		

^a^Three vessels with one nylon bag containing PFAA hay and a second nylon bag filled with PFAA-free hay

^b^Three vessels with two nylon bags with PFAA hay

^c^Six vessels contained two nylon bags filled with PFAA-free hay

^d^Limit of detection: 0.5 µg/kg

^e^Limit of detection: 0.2 µg/kg

* Ingredients: amount (g/kg dry matter), percentage, or international units (IU/kg) ash 69.1, crude protein 196.2, crude fat 42.4, crude fibre 94.8, acid detergent fibre 149.4, neutral detergent fibre 305.5, acid detergent lignin 55.7, organic matter 93.09, calcium 1.5 %, phosphorous 0.55 %, sodium 0.25 %, vitamin A 20,000 IU, vitamin D3 1600 IU

During the control period, the pH value in the fermentation vessels remained constant (Additional file 1: Figure S1A). The pH value decreased significantly when the PFAA-free hay was exchanged with the PFAA hay. The decrease in pH value was more pronounced for the vessels with high PFAA levels than for vessels with low PFAA levels (Table 2). The differences in pH value might be related to the different production level of volatile fatty acids (VFA).

**Table 2:** Fermentation characteristics of PFAA-free hay and PFAA hay with low and high PFAA levels

Item	PFAA level	PFAA-free hay^c^ (day 7–12)	PFAA hay (day 13)	SEM	*P* value^d^
pH	Low^a^	6.87 ± 0.03	6.77 ± 0.08	1.60	≤0.05
	High^b^	6.86 ± 0.02	6.69 ± 0.09	2.16	≤0.05
Redox potential, mV	Low^a^	−271 ± 19	−309 ± 30	29.7	≤0.05
	High^b^	−267 ± 24	−298 ± 27	31.9	≤0.05
Ammonia, mmol/l	Low^a^	11.77 ± 0.50	9.82 ± 1.12	1.87	0.09
	High^b^	11.81 ± 0.61	6.85 ± 0.42	0.91	≤0.05
Volatile fatty acids (VFA), mmol/day					
Total VFA	Low^a^	22.32 ± 1.00	25.90 ± 0.78	1.60	≤0.05
	High^b^	22.78 ± 0.85	28.38 ± 0.52	2.16	≤0.05
Molar proportion (%)					
Acetate	Low^a^	66.46 ± 0.50	64.03 ± 0.20	0.99	≤0.05
	High^b^	65.55 ± 1.92	61.80 ± 1.18	2.25	≤0.05
Propionate	Low^a^	25.39 ± 0.49	28.65 ± 0.26	1.25	≤0.05
	High^b^	26.46 ± 2.02	31.41 ± 1.22	2.60	≤0.05
Butyrate	Low^a^	8.15 ± 0.27	7.32 ± 0.14	0.39	≤0.05
	High^b^	8.00 ± 0.38	6.79 ± 0.10	0.56	≤0.05

^a^Three vessels with one nylon bag containing PFAA hay and a second filled with PFAA-free hay

^b^Three vessels with two nylon bags with PFAA hay

^c^Six vessels contained two nylon bags filled with PFAA-free hay

^d^Comparison of the mean was performed using *t* test

The average total VFA concentration (22.55 mmol/day) was affected after exchanging the PFAA-free hay with the low (25.90 mmol/day) and high (28.38 mmol/day) PFAA hay (Table 2). It was assumed that the higher levels of CF and nitrogen-free extracts (NfE) in the PFAA hay contribute to the increase in total VFA production. PFAA hay depressed production of acetate and butyrate and significantly increased propionate production. Higher proportions of propionate in rumen are generally observed by supplementation of readily fermentable starch-rich diets [37]. The higher level of NfE (readily fermentable carbohydrates such as starch and sugar) in the PFAA hay likely enhanced the growth of propionate producers.

During control period, the average ammonia concentration was 11.79 mmol/l, but significantly decreased in the fermentation vessels after receiving the PFAA hay (Additional file 1: Figure S1B), whereas the effect was less pronounced for the low PFAA dose (9.82 mmol/l) than for the high PFAA dose (6.85 mmol/l) (Table 2). The results indicated that ammonia was produced in a dose-related manner depending on the different protein levels of the hay.

Two electrodes used for the measurement of the redox potential showed an average result of −269 mV during the control period (Additional file 1: Figure S2). The redox potentials in the fermentation vessels decreased to −309 and −298 mV after receiving the low and high doses of PFAA hay, respectively (Table 2). It was assumed that this was associated with the increased production of propionate due to its higher demand for hydrogen.

The authors concluded that changes in fermentation characteristics were affected by the different nutrient composition of the hay, and most probably not because of the contamination with PFAAs. The constantly stable fermentation process during the whole study further indicates there were no detectable detrimental effects of PFAAs on the microbial fermentation in rumen.

### Levels in the fermentation liquids

**Fig. 1:**
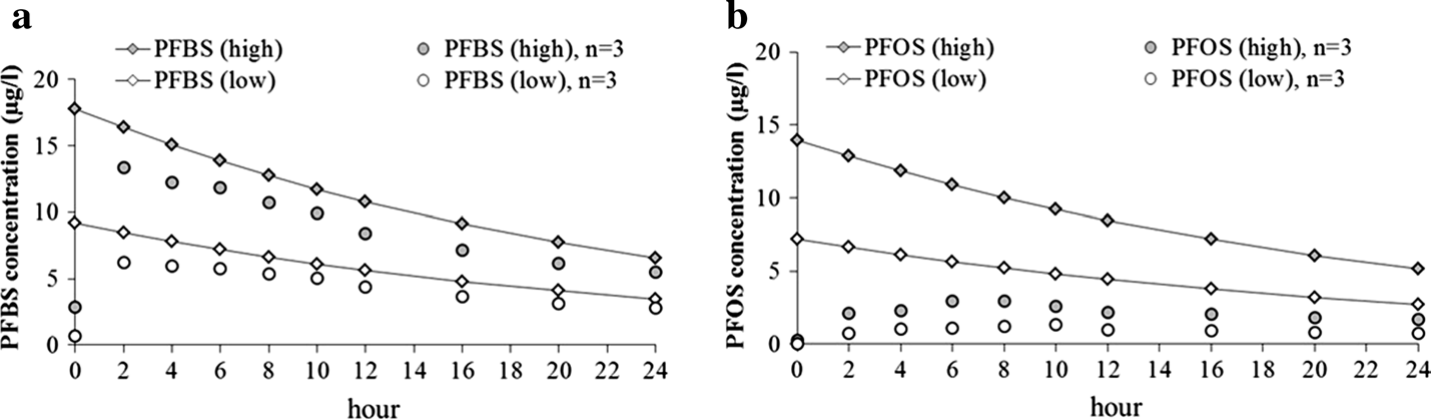
Comparison of predicted (*diamond lined*) and analyzed (*point*) concentration of PFBS (**a**) and PFOS (**b**) during 24 h fermentation

In the fermentation liquids, highest levels were found at different time points for PFBS (2 h) and PFOS (8 h), which indicates that the release of short-chain PFSAs from feed to fermentation liquid was faster (Fig. 1). No significant differences in dilution rate could be observed for PFCAs (Additional file 1: Figure S3). Different dilution rates were found between the analytical and predicted values, whereas PFOS and PFOA showed highest average differences. The effect of PFAA adhesion to the equipment surfaces were excluded by the performing an experiment B (same experimental procedure as experiment A, but under sterile conditions). In experiment B, less than 1 % of PFAAs were recovered in leachates after washing the equipment with methanol. Apart from the adhesion of PFAAs to equipment surfaces, differences between the analytical and predicted values are posited to be an effect of PFAA inclusion in biofilms formed on the RUSITEC surfaces and possibly affected the release of PFAAs into the fermenter liquid. However, this conclusion is only hypothetical because biofilms were not examined in experiment A (+MOs).

### Partitioning between liquids and feed

**Fig. 2:**
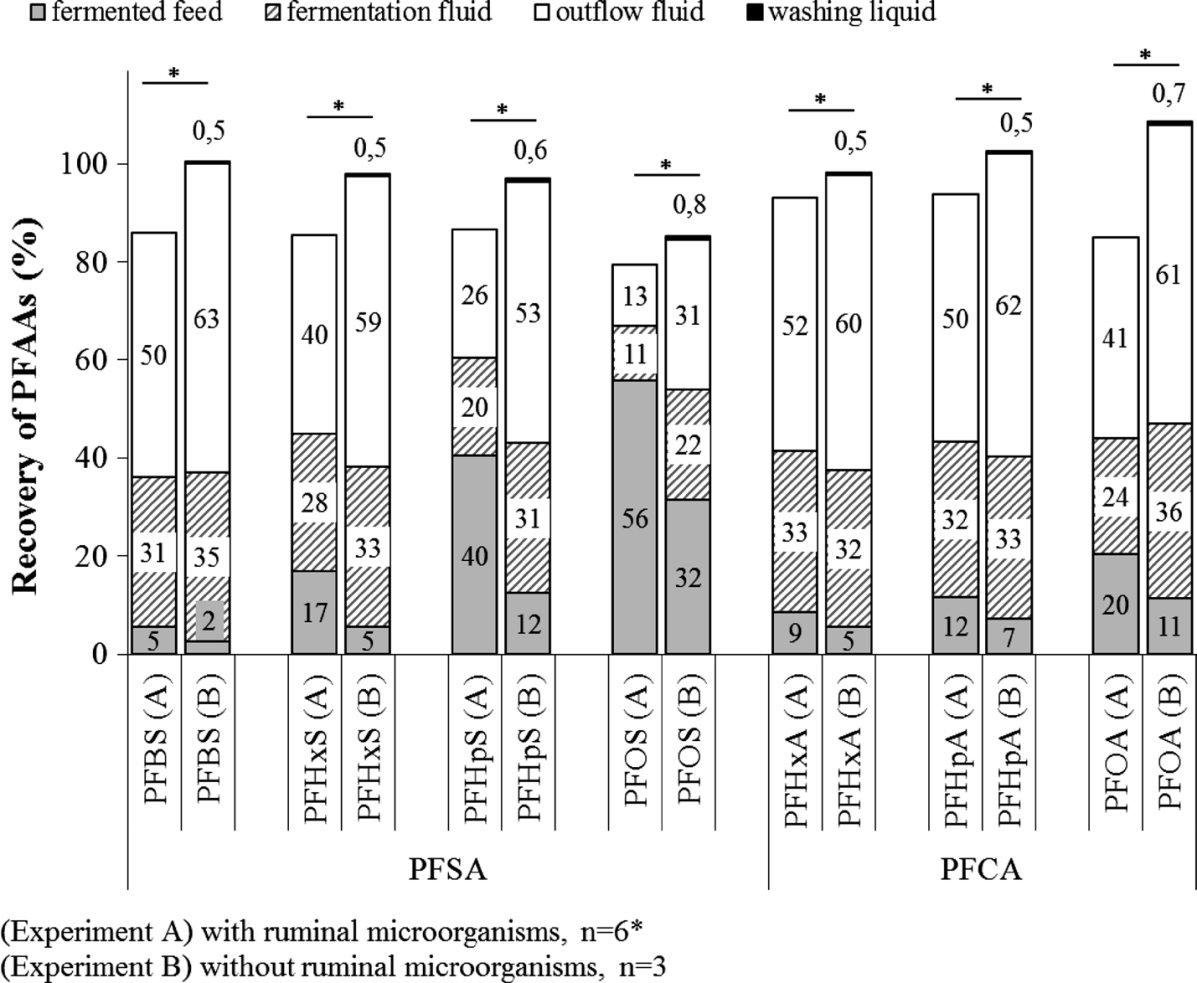
Average recovery rates of PFAAs in fermented feed, fermentation liquid, outflow fluid and the washing liquid after 24 h fermentation. *Asterisk* in experiment A, no significant differences could be found among vessels with high and low dose. Thus, comparison of the mean of experiment A and experiment B was performed after combining the results of all vessels (n = 6) in experiment A. Statistically evaluation was done by using *t* test

To calculate the proportion of PFAAs in the liquid and solid phase after fermentation, the mean concentration in each sample was referred to its initial PFAA concentration in feed (0 h) set as 100 %. The PFSA proportion in the fermentation liquids was seen to increase with decreasing chain length of the substance (Fig. 2). PFBS was higher (31 %) than the respective proportions that have been observed for PFHxS (28 %), PFHpS (20 %), and PFOS (11 %). A similar trend was observed for the PFCAs, whereas proportions of PFHxA and PFHpA did not differ significantly. The same distribution pattern but to a slightly higher extent was observed for PFSAs and PFCAs in the outflow liquids. Inverse proportions of PFAAs related to chain length were observed in the fermented feed. Regardless of PFAA dose, lowest levels in fermented feed were found for PFBS (5 %), which was three-times lower than for PFHxS (17 %) and tenfold lower than for PFOS (56 %). Levels in fermented feed increased with increasing chain length also for PFCAs, but to a lower extent (Fig. 2).

The partitioning behaviour of PFAAs is a function of their physicochemical properties [38–40]. The partitioning behaviour of PFAAs between liquid and solid phase in this study is similar to the partitioning between water and sediment in other studies [41, 42]. Ahrens and co-workers showed that short-chain PFCAs (*C* ≤ 7) could be found solely in pore water, while the longer PFCAs were present in sediment [41]. The hydrophobic character of perfluoroalkyl acids is stronger with increasing chain length [43]. Thus, longer-chain PFAAs have stronger hydrophobic interactions with the organic matter [39, 40]. Comparison of PFSAs and PFCAs with the same chain length indicated that partitioning between liquids and feed is also influenced by functional groups. The stronger absorption of the PFSAs to organic matter indicates that the sulfonate group exhibits lower water solubility and stronger hydrophobicity than the carboxylate group [40].

Experiment B (−MOs) showed significant differences of PFAAs proportion in the fermentation liquids and feed compared to experiment A (+MOs) (Fig. 2). This effect can be explained with the fact that 70–80 % of the total microbial population in the rumen is attached to feed particles [44]. Because of the attachment of MOs to feed particles in experiment A (+MOs), less PFAAs could dissolve in the fermentation liquids, thus accounting for the higher levels in fermented feeds compared to experiment B (−MOs).

### Total recovery

To calculate the recovery of PFAAs, the mean concentration in each sample was referred to its initial PFAA concentration in feed (0 h) and set as 100 %. In experiment A (+MOs), the statistical evaluation showed no differences in PFAA recovery among the vessels with high and low PFAA doses (data not shown). For this reason, the results of PFAA recovery with low and high doses of experiment A (+MOs) were combined and statistically compared with the results of PFAA recovery of experiment B (−MOs). Figure 2 shows the percentages of PFAAs recovered in the solid phase (fermented feed) and the liquid phases (fermentation liquid, outflow liquid) for the samples of experiment A and experiment B.

In experiment A (+MOs), total recovery was almost constant for PFCAs and PFSAs up to carbon chain-lengths of ≤C7 of 93–94 % and 85–87 %, respectively. Lowest total recovery was examined for PFOA (85 %) and PFOS (80 %). Without the presence of MOs, total recovery was found to be 98 % for PFHxA, 103 % for PFHpA and 109 % for PFOA which was significantly higher compared to the total recovery of experiment A (Additional file 1: Table S1). Significantly higher levels were also found for PFSAs with lowest total recovery for PFOS (PFBS 101 %, PFHxS 98 %, PFHpS 97 %, PFOS 85 %). However in experiment A (+MOs), the missing percentages of PFAAs to total recovery of 100 % could not be explained by the standard deviation. The experimentally calculated standard deviation for recovery replicates was very low (5.6 % for PFHpS and ≤2.8 % for all other substances). The interpretation of the results is markedly complicated for PFOS because the total recovery resulted in a PFOS disappearance of 15 % in the absence of MOs. Overall, by using RUSITEC, the mechanism of PFAA disappearance in the presence of MOs remains unclear.

In studies on biochemical degradation of perfluorinated compounds in sewage sludge, disappearance of PFOS and PFOA could only be observed under anaerobic conditions with redox potentials below −300 mV [28]. However, since neither fluorine ions nor transformation products could be detected, it remained unclear if disappearance of PFOA and PFOS was a microbially-mediated degradation leading to mineralization. Ochoa-Herrera and co-workers studied the PFOS degradation under reductive conditions without presence of MOs and by the use of cobalamin and titanium(III)citrate and observed significant reduction of PFOS [30]. They suggested that both molecules play a key role in the kinetics of PFOS degradation. Based on chromatography analysis, the researchers noticed that branched PFOS isomers, but not linear PFOS isomers disappeared during the reductive defluorination. However, in the current study, only the linear PFOS isomers were quantified, so the authors can rule out that disappearance of PFOS of 15 % was affected by the degradation of branched chain PFOS isomers.

In the literature, researchers also discuss whether the reductive defluorination of PFOA might be a cometabolic reaction carried out by mixed microbial communities [29]. A cometabolic reaction is defined as a breakdown of a contaminant by an enzyme with a cofactor that is produced during microbial metabolism of another compound under anaerobic conditions [45]. In fact, the rumen includes diverse MOs producing a complex mixture of enzymes and metabolites. In anaerobic MOs, some dehalogenating enzymes have been characterized. For the catalytic activity of the dehalogenating enzymes, the involvement of cobalamin has also been shown in MOs [29]. Fermentation in rumen is known to be a primary source of cobalamin for ruminants. Several organisms were identified to be responsible for cobalamin production in the rumen, e.g. *Selenomonas ruminantium* and *Peptostreptococcus elsdenii* [33]. The total mass of MOs in the rumen consists of bacteria (10^11^/g), protozoa (10^4^/g) and fungi (10^5^/g) [46] which are mutually involved in complex metabolic processes, and theoretically provide a large gene pool, e.g. for recruiting specific enzymes that can carry out a cometabolic reaction as a critical step for biodegradation [47]. Furthermore, there are optimal reductive conditions for MOs in rumen (−300 mV) by VFA production through fermentation of structural carbohydrates. Taking into account the commonly available conditions in the rumen, such a cometabolic reaction might explain the indirect microbiologically driven disappearance of PFAAs in rumen. Unfortunately, the ultimate proof for the degradation of PFAAs by MOs was not provided because an analysis of fluoride ions or transformation products was not performed.

Available literature data about PFAAs biodegradation generally failed to provide results of fluoride ion loss as indication for PFAAs mineralization [28, 29, 48, 49]. Although the reductive defluorination is supposed to be energetically favourable for bacteria under anaerobic conditions, it may be hindered by the high strength of the C-F bond [18, 31, 50]. This is the reason why PFAAs are generally considered to be microbiologically resistant to degradation.

## Conclusion

This study shows that the partitioning of PFAAs between the solid and liquid phase in the rumen is influenced by their physicochemical properties, but also by the presence of ruminal MOs having an effect on the dilution rate of PFAAs from the feed into the fermentation liquid. This conclusion is confirmed by the lower recovery rates of PFAAs in fermented feed in the presence of ruminal MOs.

For PFAA disappearance in experiment A (+MOs), the potential effects of a cometabolic reaction as an indirect microbiologically-mediated PFAA degradation mechanism was discussed on the one hand and on the other the formation of biofilms as a sink for PFAAs in a microbial system. The mechanisms for PFAA disappearance in RUSITEC still remain inconclusive, since analysis of fluoride ions or transformation products was not performed.

PFAAs are accumulated more to a larger extent in muscle tissues of monogastrics compared to ruminants [1, 2]. In the context of consumer risk assessment, the hypothesis was formulated that this may be due to a disappearance (low recovery) as a consequence of the complex microbial ecosystem in the rumen. Using the RUSITEC we found indications for a small but statistically significant disappearance of PFOS (15 %). This is however not enough to explain the said difference between monogastric and ruminants (40–49 % vs <0.001–43 % accumulation of ingested PFAAs in muscle tissue). Beyond this, an interesting question is whether biofilms are an important sink for PFAAs in a microbial system.

## Methods

### Hay cultivation and preparation

PFAA contaminated hay was cultivated and harvested from a PFAA-contaminated agricultural land in Lower-Saxony, Germany. The agricultural land was contaminated by the use of a soil improver containing industrial waste with high concentrations of PFAAs. The soil improver provided by a recycling company was spread by the farmer on the agricultural land for several years before the environmental contamination became evident [51].

The second-cut PFAA hay was stored in square bales at the Federal Institute for Risk Assessment (BfR) and analyzed for PFAAs concentration at the Chemical and Veterinary Analytical Institute in Münster (Table 1). The PFAA-free hay (1st cut) was cultivated and harvested from a PFAAs unpolluted farmland in Lower-Saxony, Germany, provided by the Department Institute of Physiology of the University of Veterinary Medicine Hannover, Foundation. For sample preparation, PFAA-free hay and PFAA hay were cut with a sheep shearing machine into small pieces of 0.5–1 cm. Thereafter, the hay were divided homogeneously into 10 g samples by a rotary sample divider.

### Experimental procedure and sampling

#### Experiment A (with ruminal MOs)

The RUSITEC experiment with six fermentation vessels was carried out similar as described previously [52]. Rumen inoculum was collected in the morning (3 h after feeding) from a ruminal fistulated Hinterwälder cow (13 years old, 650 kg body weight, non-lactating). Donor animal received hay and concentrate of 9 kg and 200 g per day, respectively. Concentrate is usually used as part of feed for ruminants. As this in vitro study should represent normal feeding conditions it was necessary to add concentrate provided by DEUKA Schaffutter, Erfurt, Germany. At the beginning of the experiment, each fermentation vessel was inoculated with 80 g of solid rumen content and 700 ml rumen liquid.

The experiment consisted of an equilibration period of 6 days and a consecutive control period of 6 days followed by an experimental period of 24 h. During equilibration and control period nylon bags (11.5 × 6.5 cm, pore size 150 µm) comprised 8 g of PFAA-free hay and 2 g concentrate. After 24 h of equilibrium period, one nylon bag in each vessel was replaced with a nylon bag containing PFAA-free hay and concentrate. Throughout the overall experimental period the retention time of each nylon bag was adjusted to 48 h, i.e. the nylon bags were exchanged alternately at 24 h intervals. At the beginning of the experimental period, three fermentation vessels received two nylon bags with 8 g PFAA hay and 2 g concentrate (high dosage), whereas the remaining three fermentation vessels received one nylon bag containing 8 g PFAA hay and 2 g concentrate and a second nylon bag filled with 8 g PFAA-free hay and 2 g concentrate (low dosage).

For simulation of saliva secretion each fermentation vessel was connected to a fermenter pump about which buffer solution was constantly infused (0.7 l/day). The dilution rate of PFAAs in the fermentation liquids was determined during experimental period by relating the initial PFAA concentration in feed to the turn over rate of the fermentation fluid in each vessel. The time course of the diluted PFAA in the fermentation liquid (*C*_d_) was calculated using equation:

$$C_{\text{d}} = a_{0} \cdot {\text{e}}^{{{-}kt}}$$

where *a*_0_ is the initial concentration of the PFAA (µg/l), –*k* is the dilution value estimated by division of fermentation vessel volume (l) and turn over rate of fermenter pump (l/day), and *t* is the duration of the experiment expressed in days. The buffer solution (28 mmol/l NaCl; 7.69 mmol/l KCl; 0.5 mmol/l 1 N HCl; 0.22 mmol/l CaCl_2_∙2H_2_O; 0.63 mmol/l MgCl_2_∙6H_2_O; 5 mmol/l NH_4_Cl; 10 mmol/l Na_2_HPO_4_∙12H_2_O; 10 mmol/l NaH_2_PO_4_∙H_2_O; 97.90 mmol/l NaHCO_3_) was infused continuously to reach a liquid turnover of once a day. Effluents of vessels were collected in air tight glass flasks which were stored on ice to inhibit microbial fermentation. The fermentation gases were simultaneously collected in gas-tight bags (Plastigas, Linde AG, München, Germany). PFAA-free hay, PFAA hay, concentrate, distilled water used for buffer preparation and nylon bags were analyzed for contents of PFAAs. In addition, chemical composition of PFAA-free and PFAA hay was determined (Table 1).

Determination of pH and redox potentials were done using the Digital-pH-Meter 646 (Knick GmbH & Co. KG, D-14163 Berlin) and the redox-electrode InLab^®^ RedoxPro (Mettler-Toledo GmbH, D-35353 Gießen), respectively. The measurements were carried out throughout the experiment to monitor the anaerobic status of the system (redox potential normally varies between −100 and −300 mV) [53, 54] and to ensure adequate environmental conditions (pH between 5.5 and 7.0) for microbial survival.

Concentration of VFA in effluents was analyzed daily as described previously [55]. VFA production was determined by multiplying concentrations and volumes of the effluent.

For analyzes of ammonia concentrations 1 ml of the effluents was centrifuged at 4600 g for 10 min followed by mixing 50 µl of the supernatant with 5 ml phenol solution (106 mM phenol, 0.17 mM sodium nitroprusside dihydrate) and 5 ml sodium hypochlorite solution [1 % (v/v) sodium hypochlorite; 125 mM NaOH]. After an incubation step at 60 °C in a water bath for 10 min, concentrations of ammonia were determined photometrically at 546 nm in a spectrometer (DU 640, Beckman Coulter GmbH, Krefeld, Germany) using a NH_4_Cl standard solution (5 mM).

During the experimental period, 40 ml of fermentation liquid were retrieved from each vessel for determination of PFAA concentration at following points after exchanging feed: 0, 2, 4, 6, 8, 10, 12, 16, 20, 24 h. In addition, the infused buffer volume was recorded at each point. Counteracting the continuous loss of fermentation liquid by sampling, the deviating volume was refilled from further six fermentation vessels which were treated equally.

At the end of the experiment both nylon bags were removed, dried at 65 °C and analyzed for concentration of PFAAs. Finally, volume of fermentation liquid was measured.

#### Experiment B (without ruminal MOs)

A second experiment with three fermentation vessels was conducted under sterile conditions. Therefore, the nylon bags and the feed were sterilized in an autoclave by subjecting them to high pressure saturated steam at 103 and 121 °C, respectively. At the beginning of the experiment, each fermentation vessel was filled with two nylon bags containing 8 g PFAA hay and 2 g concentrate and 750 ml buffer solution (for composition see “Experiment A”). For PFAA analysis, 50 ml of liquid was retrieved from each fermentation vessel. The buffer solution was infused continuously to reach a liquid turnover of once a day.

After 24 h, samples of 50 ml were taken from the liquid of each fermentation vessel and from the effluents and were analyzed for PFAA concentrations. In addition, total liquid volume in fermentation vessels and volume of effluents were measured. Both nylon bags were dried at 65 °C and analyzed for PFAA concentrations.

Subsequently, each fermentation vessel was washed with 100 ml of methanol solution (25 %) to dissolve adsorbed PFAAs from vessel surfaces. The washing solution was collected and analyzed for PFAA concentrations.

### PFAA analysis

#### Sample preparation

Because of possible interferences between PFAA and proteins, matrix specific sample preparations were performed. For sample storage and sample preparation, only vessels made of polypropylene (PP) were used and served as blank in each series of PFAA analyzes. 1–5 g feed samples were extracted with methanol and an aliquot of this solution was diluted with water (VDLUFA-Method) [56]. About 5 ml (10 ml) methanol were added to 10 ml (20 ml) RUSITEC sample and afterwards the pH-value was adjusted to 4.5–5.5. All sample solutions were purified and concentrated using solid phase extraction on an OasisWAX column (150 mg/6 ml) [57]. The final extract was reconstituted in 100–1000 µl methanol/water (50/50, v/v), depending on the expected concentration.

#### Measurement

The purified solutions were measured using high-performance liquid chromatography (HPLC) with tandem mass spectrometry (MS/MS) in negative ion multiple reaction monitoring (MRM) mode. The separation was performed on an Agilent 1200 SL HPLC-System. A mixture of 2 mM ammonium acetate/acetonitrile (95/5, v/v) and a mixture of methanol/acetonitrile (40/60, v/v) were used as solvents in a gradient elution. MS/MS-detection was performed with an Agilent 6460 triple quadrupole mass spectrometer equipped with an electrospray interface (ESI) operating in the negative ion mode. The MRM-settings are published elsewhere [58]. In each sample sequence a blank-sample and a none-contaminated sample, which was spiked with isotope labeled PFHxS, PFOA and PFOS, was measured. The recovery was between 90 and 100 %. The analytical results were corrected for recovery rates. The a priori relative standard deviations were about 20 % for concentrations near the limit of quantification and 10–15 % for higher concentrations.

#### Quantification

Quantification was performed with isotope labeled standards and a seven-point calibration curve. ^18^O-PFHxS and later ^13^C-PFHxS were used as internal standards for PFBS and PFHxS. ^13^C-PFOA was used as internal standard for PFOA and ^13^C-PFOS for PFOS. The internal standards were added at the beginning of the sample preparation. The limit of detection was defined as the signal to noise ratio of 3:1 of the qualifier ion. The limit of quantification is defined as the concentration on which a substance is identified unequivocally and quantified with a relative standard deviation of 20 % or lower. The measurement range was between 0.2 and 100 µg/l. In the case of PFOS, it could be ensured that only the linear substance was quantified. Due to the fact that standard substances of branched PFHpS and PFHxS were not available, it was not possible to ascertain that all the branched substances are separated completely from the linear ones. Only peaks with the same retention time as the linear substances were integrated. The ratios of the two most intensive MRM-transitions of the integrated PFHpS and PFHxS are the same as in the linear standard substance. Due to the finding that the transitions of the branched isomers differ from the transitions of the linear substances in the case of PFOS, this is an indication that the uncertainty caused by the branched isomers is small for PFHpS and PFHxS. The PFCA concentrations in the analyzed matrices were very low, so that a differentiation between the linear and the branched substances was not possible. It is described in literature that 78 % of the PFOA manufactured by the 3 M Company is linear [59, 60]. The ratios of the two most intensive MRM-transitions of the integrated PFCA were the same as in the linear standard substances. So it can be assumed that also in the case of PFCA the potential uncertainty caused by the branched isomers is small.

A short HPLC-column was placed as a pre-column between purge valve and autosampler to separate background PFCA and PFAS from the analytes of the samples. An injector program was used to minimize potential cross-contamination from heavily contaminated samples as far as possible. Interferences of PFOS with taurodeoxycholic acid are excluded, because both substances are separated chromatographically and furthermore the relation of the two most intensive transitions of PFOS in comparison to a standard solution was used to check possible interferences. Taurodeoxycholic acid does not show the *m*/*z* transition 499 to 99, which is specific for PFOS. The analytical method is described in detail by Ehlers [61].

#### Statistical evaluations

Data were processed using the statistic program SPSS1201 version 7.0.1.4. Effects on fermentation parameters were determined using *t* test if normal distribution of data was given. If not, Kruskal–Wallis test was used. For testing normal distribution Kolmogorov–Smirnov test was used. The homogeneity of variances was tested using the Levene’s test. The significant differences of redox potential between each time point were determined by one-way analysis of variance. Here, the Dunett-T3 test was used when homogeneity of variance was not given. Comparison of the mean PFAA levels between samples of experiment A and experiment B of the same matrix was performed using *t* test, whereas normal distribution was assumed. The data are reported as the mean ± standard deviation. All differences were considered to be significant when *P* ≤ 0.05.

## Additional file

10.1186/s12302-015-0063-4 Can perfluoroalkyl acids biodegrade in the rumen simulation technique (RUSITEC)?

## Abbreviations

APFO: ammonium perfluorooctanoate
CF: crude fiber
CP: crude protein
DM: dry matter
ESI: electrospray interface
HPLC: high-performance liquid chromatography
MOs: ruminal microorganisms
MRM: multiple reaction monitoring
mV: millivolt
NfE: nitrogen free extracts
PFAAs: perfluoroalkyl acids
PFAS: per- and polyfluoroalkyl substances
PFBS: perfluorobutane sulfonic acid
PFCAs: perfluoroalkyl carboxylic acids
PFHpA: perfluoroheptane carboxylic acid
PFHpS: perfluoroheptane sulfonic acid
PFHxA: perfluorohexane carboxylic acid
PFHxS: perfluorohexane sulfonic acid
PFOA: perfluorooctanoic acid
PFOS: perfluorooctane sulfonic acid
PFSAs: perfluoroalkyl sulfonic acids
POP: persistent organic pollutant
PP: polypropylene
REACH: European chemicals regulation, EC No. 1907/2006
RUSITEC: rumen simulation technique
SVHC: substances of very high concern
SEM: standard error of the mean
US EPA: United States environmental protection agency
VFA: volatile fatty acids
