# Supplementary material for: Can perfluoroalkyl acids biodegrade in the rumen simulation technique (RUSITEC)?
Source: Environ Sci Eur. 2015 Nov 23;27(1):30. doi: 10.1186/s12302-015-0063-4 (PMC5044947; doi:10.1186/s12302-015-0063-4)
Supplement: Supplementary file 1 — 10.1186/s12302-015-0063-4 Can perfluoroalkyl acids biodegrade in the rumen simulation technique (RUSITEC)? [file 12302_2015_63_MOESM1_ESM.doc]

**Can Perfluoroalkyl Acids Biodegrade in the Rumen Simulation Technique (RUSITEC)?**

Authors: J. Kowalczyk^a^,*,

S. Riede^b^,

H. Schafft^a^,

G. Breves^b^,

M. Lahrssen-Wiederholt^a^

^a^ Federal Institute for Risk Assessment, Max-Dohrn-Str. 8-10, 10589 Berlin

^b^ Department Institute of Physiology, University of Veterinary Medicine Hannover, Foundation, Bischofsholer Damm 15, 30173 Hannover

E-mail: Janine.Kowalczyk@bfr.bund.de

Journal: Environmental Sciences Europe

**Table of content**

[Figure S 1 Effects of PFAA-free hay and PFAA hay on the pH (A) and the ammonia production (B) 2](#__RefHeading___Toc428514987)

[Figure S 2 Effects of PFAA-free hay and PFAA hay on redox potential (mV) 3](#__RefHeading___Toc428514988)

[Figure S 3 Comparison of predicted (diamond lined) and analyzed (point) concentration of PFHxA (A), PFHpA (B), PFOA (C), PFHxS (D), and PFHpS (E) during 24 h fermentation 4](#__RefHeading___Toc428514989)

[Table S 1 Mean ± standard deviation of PFAAs recovered in experiment A (+MOs) and experiment B ( MOs) after 24 h fermentation 5](#__RefHeading___Toc428514990)

[Table S 2 PFAA levels in the feed materials and destilled water 6](#__RefHeading___Toc428514991)

**
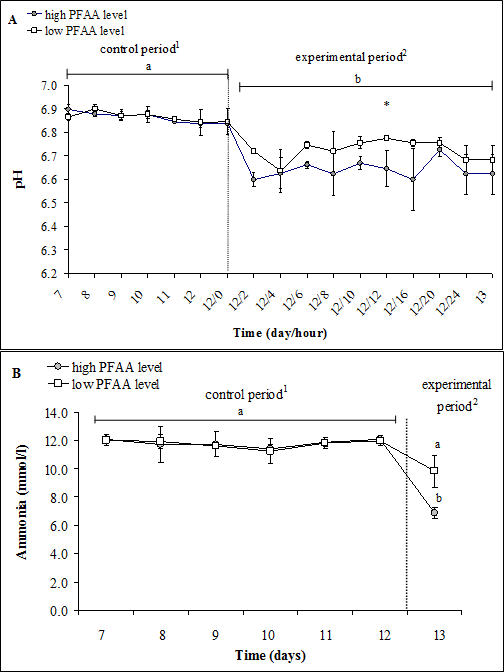
**

^1^ Six fermentation vessels contained two nylon bags filled with PFAA-free hay and concentrate, respectively.

^2^ Three of six fermentation vessels received two nylon bags with PFAA hay and concentrate, whereas the other three fermentation vessels received only one nylon bag containing PFAA hay and concentrate and a second filled with PFAA-free hay and concentrate.

^a,b^ Different subscripts indicate significant differences between control period and experimental period (P ≤ 0.05)

* Significant differences between high and low PFAA levels (P ≤ 0.05)

Figure S 1 Effects of PFAA-free hay and PFAA hay on the pH (A) and the ammonia production (B)

^1^ Six fermentation vessels contained two nylon bags filled with PFAA-free hay and concentrate, respectively.

^2^ Three of six fermentation vessels received two nylon bags with PFAA hay and concentrate, whereas the other three fermentation vessels received only one nylon bag containing PFAA hay and concentrate and a second filled with PFAA-free hay and concentrate.

^*^ Differences between high and low PFAA-levels were not significant for each time point.

^a,b,c,d^ Different subscripts indicate significant differences between the samples of each time point (P ≤ 0.05).

Figure S 2 Effects of PFAA-free hay and PFAA hay on redox potential (mV)


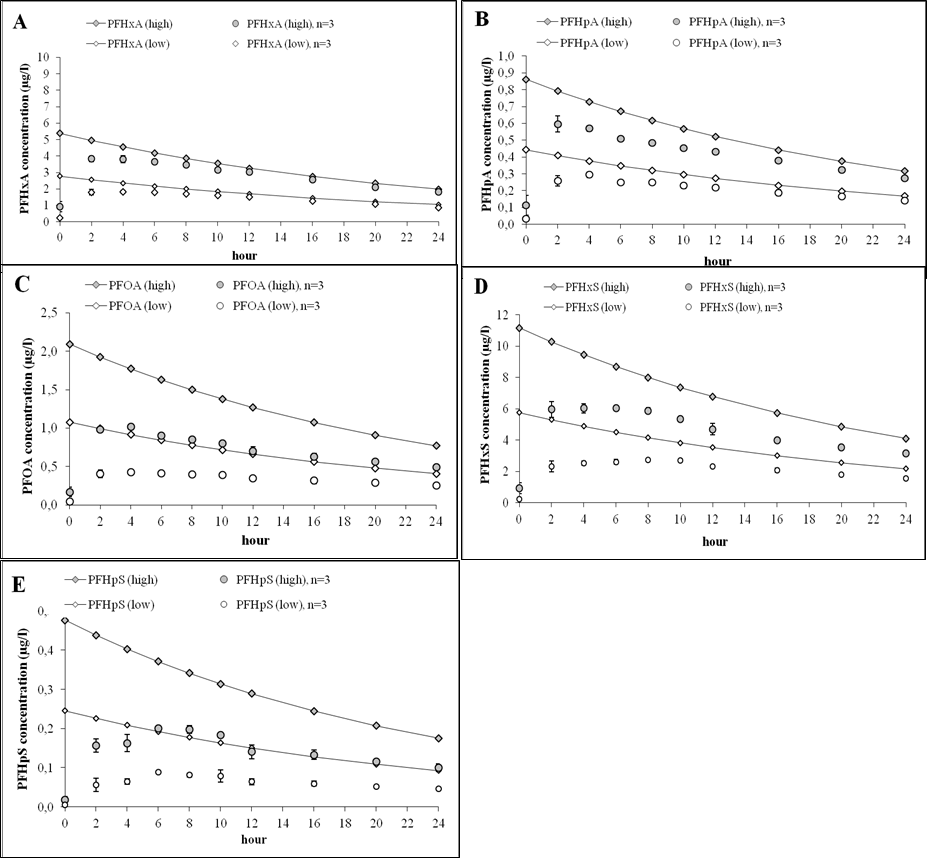


Figure S 3 Comparison of predicted (diamond lined) and analyzed (point) concentration of PFHxA (A), PFHpA (B), PFOA (C), PFHxS (D), and PFHpS (E) during 24 h fermentation

Table S 1 Mean ± standard deviation of PFAAs recovered in experiment A (+MOs) and experiment B ( MOs) after 24 h fermentation

| Recovery (%) | **PFBS** | **PFHxS** | **PFHpS** | **PFOS** | **PFHxA** | **PFHpA** | **PFOA** |
| --- | --- | --- | --- | --- | --- | --- | --- |
| **Fermentation liquid** |  |  |  |  |  |  |  |
| Experiment A^1^ | 31 ± 0,8 | 28 ± 1,2 | 20 ± 2,0 | 11 ± 1,5 | 33 ± 1,9 | 32 ± 1,9 | 24 ± 1,3 |
| Experiment B^2^ | 35 ± 1,0 | 33 ± 1,9 | 31 ± 1,8 | 22 ± 1,4 | 32 ± 1,5 | 33 ± 1,5 | 36 ± 2,0 |
| *P*-value^3^ | <0.001 | 0.002 | <0.001 | <0.001 | 0.606 | 0.311 | <0.001 |
| **Outflow liquid** |  |  |  |  |  |  |  |
| Experiment A^1^ | 50 ± 1,9 | 40 ± 3,8 | 26 ± 4,3 | 13 ± 2,5 | 52 ± 1,0 | 50 ± 2,1 | 41 ± 4,3 |
| Experiment B^2^ | 63 ± 1,7 | 59 ± 1,9 | 53 ± 1,9 | 31 ± 0,4 | 60 ± 0,7 | 62 ± 1,2 | 61 ± 2,2 |
| *P*-value^3^ | <0.001 | <0.001 | <0.001 | <0.001 | <0.001 | <0.001 | <0.001 |
| **Fermented feed** |  |  |  |  |  |  |  |
| Experiment A^1^ | 5 ± 1,0 | 17 ± 2,9 | 40 ± 5,0 | 56 ± 3,6 | 9 ± 1,0 | 12 ± 1,5 | 20 ± 3,2 |
| Experiment B^2^ | 2 ± 0,1 | 5 ± 0,1 | 12 ± 0,8 | 32 ± 1,7 | 5 ± 0,1 | 7 ± 0,1 | 11 ± 0,4 |
| *P*-value^3^ | 0.002 | <0.001 | <0.001 | <0.001 | 0.002 | 0.001 | 0.002 |
| **Total** |  |  |  |  |  |  |  |
| Experiment A^1^ | 86 ± 2,1 | 85 ± 2,8 | 87 ± 5,6 | 80 ± 2,2 | 93 ± 2,5 | 94 ± 2,2 | 85 ± 4,5 |
| Experiment B^2^ | 101 ± 2,5 | 98 ± 2,0 | 97 ± 1,6 | 85 ± 1,7 | 98 ± 0,9 | 103 ± 0,5 | 109 ± 0,8 |
| *P*-value^3^ | <0.001 | <0.001 | 0.019 | 0.005 | 0.013 | <0.001 | <0.001 |

^1^ Sample size of experiment A: n=6; ^2^ Sample size of experiment B: n=3

^3^ In experiment A, no significant differences could be found among vessels with high and low dose. Thus, comparison of the mean of experiment A and experiment B was performed after combining the results of all vessels (n=6) in experiment A. Statistically evaluation was done by using *t* test.

Table S 2 PFAA levels in the feed materials and destilled water

|  | **PFBS** | **PFHxS** | **PFHpS** | **PFOS** | **PFHxA** | **PFHpA** | **PFOA** |
| --- | --- | --- | --- | --- | --- | --- | --- |
| Concentrate^1^ | <LOD | <LOD | <LOD | <LOD | <LOD | <LOD | <LOD |
| PFAA-free hay^1^ | <LOD | <LOD | <LOD | <LOD | <LOD | <LOD | <LOD |
| Distilled water for buffer preparation^2^ | <LOD | <LOD | <LOD | <LOD | <LOD | <LOD | <LOD |

^1^Limit of detection (LOD): 0.2 µg/kg, except for PFBS, PFHxA: 0.5 µg/kg; ^2^ LOD: 0.001 µg/l
